# Molecular Docking of Potential Inhibitors of Broccoli Myrosinase

**DOI:** 10.3390/molecules23061313

**Published:** 2018-05-30

**Authors:** J. Román, A. Castillo, A. Mahn

**Affiliations:** 1 Doctorado en Ciencia y Tecnología de Alimentos, Facultad Tecnológica, Universidad de Santiago de Chile, Obispo Manuel Umaña 050 Estación Central, Santiago 9170019, Chile; juan.romana@usach.cl; 2 Departamento de Biología, Facultad de Química y Biología, Universidad de Santiago de Chile, Avenida Libertador Bernardo O’Higgins 3363, Estación Central, Santiago 9170019, Chile; antonio.castillo@usach.cl; 3 Departamento de Ingeniería Química, Facultad de Ingeniería, Universidad de Santiago de Chile, Avenida Libertador Bernardo O’Higgins 3363, Estación Central, Santiago 9170019, Chile

**Keywords:** thioglucosidase inhibitors, glucosinolates, sulforaphane, amygdalin, arbutin

## Abstract

Glucosinolates are secondary metabolites occurring in *Brassicaceae* plants whose hydrolysis may yield isothiocyanates, widely recognized as health-promoting compounds. Myrosinase catalyzes this conversion. The chemical mechanism involves an unstable intermediary (thiohydroxamate-*O*-sulfonate) that spontaneously decomposes into isothiocyanates or other non-bioactive compounds depending on pH and cofactors. At acidic pH, non-bioactive compounds such as nitriles and thiocyanates are formed, while at neutral pH isothiocyanates are obtained. Broccoli myrosinase has been poorly studied so far. Recently, its amino acidic sequence was elucidated, and a structural model was built. The aim of this work was to study the molecular interaction of broccoli myrosinase with different ligands at acidic pH to propose possible inhibitors that prevent formation of undesirable compounds at acidic pH, and that at neutral pH dissociate from the enzyme, allowing formation of isothiocyanates. The interaction between broccoli myrosinase and 40 ligands was studied by molecular docking simulations. Both the enzyme and each inhibitor were set at pH 3.0. Amygdaline and arbutin showed the highest affinity to broccoli myrosinase in this condition. The residues that stabilize the complexes agree with those that stabilize the substrate (Gln207, Glu429, Tyr352, and Ser433). Accordingly, amygdaline and arbutin would perform as competitive inhibitors of myrosinase at pH 3.0.

## 1. Introduction

**Figure 1:**
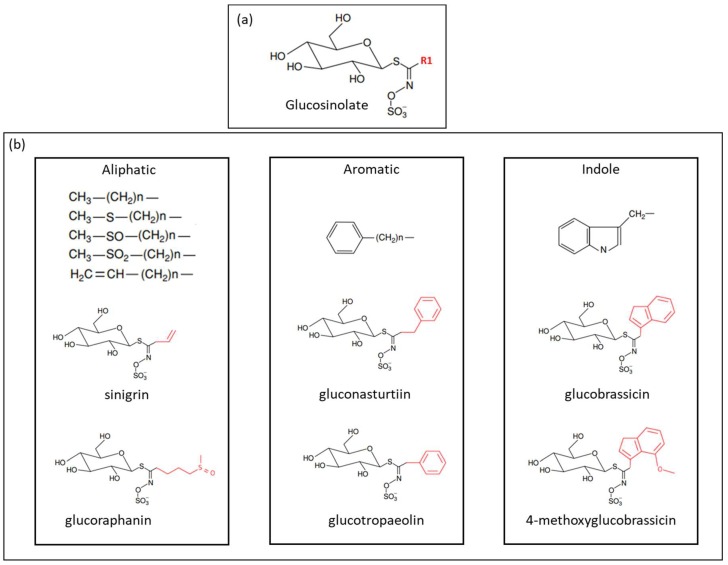
(**a**) General structure of glucosinolates, where R1 denotes the amino acidic-derived side chain; and (**b**) classification of glucosinolates according to the side chain (R1), and some examples of glucosinolates found in *Brassicaceae* vegetables (adapted from Holst et al. [5]).

Glucosinolates (GSL) are nitrogen- and sulfur-containing secondary metabolites, mainly found in *Brassicaceae* [1,2]. Approximately 130 glucosinolates have been identified. Their chemical structure consists of a β-d-glucopyranose residue linked to a hydroxylamine sulfate ester by sulfur bridge, as well as an amino acid-derived R-group, which can be aliphatic, aromatic, or indole [3,4,5]. Figure 1 shows the chemical structure of GSL.

**Figure 2:**
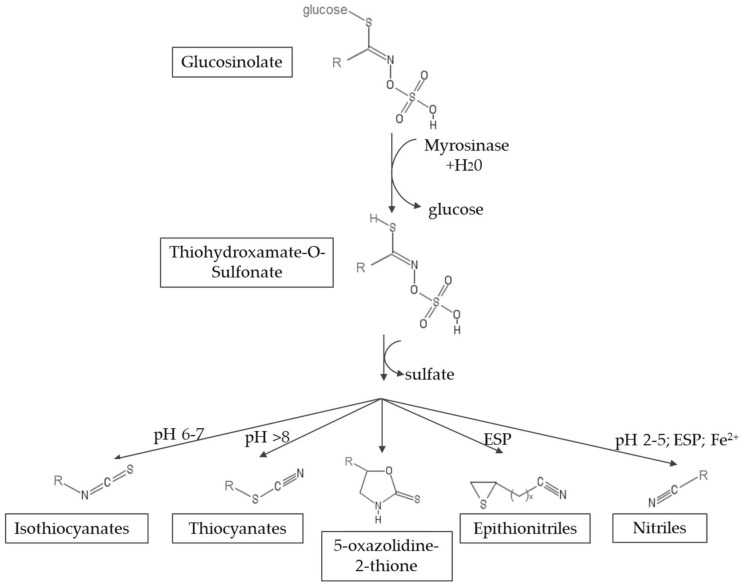
Mechanism of hydrolysis of glucosinolates by myrosinase. ESP, epithiospecifier protein (adapted from Latté et al. [2]).

Myrosinase (thioglucosidase glucohydrolase, EC 3.2.1.147) is a glycoprotein that catalyzes the hydrolysis of glucosinolates [6,7]. The hydrolysis leads to the formation of an unstable aglycone intermediate (thiohidroxamate-*O*-sulfonate), glucose and sulfate. This aglycone undergoes a spontaneous non-enzymatic Lossen rearrangement to yield isothiocyanates (ITCs), thiocyanates, nitriles, oxazolidinethiones and epithionitriles, depending on the structure of the GSL and the chemical conditions, such as pH, availability of ferrous ions and the presence of myrosinase interacting proteins [8]. Figure 2 shows a scheme of the reaction mechanism. The hydrolysis products that result from myrosinase activity on glucosinolates form part of the defense system of the plant against the attack of microorganisms, insects and herbivores, since they have insecticidal, fungicidal and bactericidal properties [9,10]. Among the hydrolysis products, isothiocyanates are of great interest because of their health promoting properties. On the contrary, nitriles have been associated with hepatic damage, liver hemorrhage and renal megalocytosis [11,12].

Sulforaphane, an isothiocyanate found mainly in broccoli, has anticancer and antimicrobial activity, providing protection to cells against exogenous or endogenous carcinogenic intermediates [13,14]. It also has demonstrated bactericidal effect against *Helicobacter pylori* [7]. Sulforaphane comes from the hydrolysis of glucoraphanin, which is the most abundant GSL in broccoli, and is scarce in other *Brassicaceae* family members. Recently, attention has been set on maximizing sulforaphane content in broccoli-derived foods through different food processing methods [15,16] to exploit the health properties of this isothiocyanate. However, the chemical instability of sulforaphane impairs its bioavailability. Moreover, after the intake of GSL, given the acidic pH and the presence of Fe^+2^ in stomach, the main products that come from GSL hydrolysis are nitriles [17]. Therefore, to improve the bioavailability of sulforaphane and other isothiocyanates, and minimize the formation of nitriles, we propose that myrosinase can probably be inhibited by small molecules that bind reversibly to the active site of the enzyme at acidic pH, thus preventing the formation of undesirable products. Then, the aim of this work was to investigate the molecular interaction of broccoli myrosinase with different ligands that have potential as pH-dependent myrosinase inhibitors.

Broccoli myrosinase has been poorly studied so far. This enzyme was purified for the first time by Mahn et al. [18], and a preliminary characterization was reported. Recently, the cDNA nucleotide sequence of broccoli myrosinase was determined (Genbank ID: MF 461331); its amino acid sequence was deduced; and a three-dimensional model of its monomer was built (PMDB ID: 00811093) [19]. No studies about the molecular interaction of broccoli myrosinase and ligands other than the substrate are available so far. In this work, we investigated the molecular interaction of broccoli myrosinase with 40 ligands at acidic pH to propose a molecule that acts as reversible inhibitor of the enzyme. The stability of the complexes was compared with the stability of myrosinase-substrate complexes. Besides, the effect of pH on myrosinase activity was studied to select the pH value at which conduct the molecular docking simulations.

## 2. Results

### 2.1. Effect of pH on Myrosinase Activity

**Figure 3:**
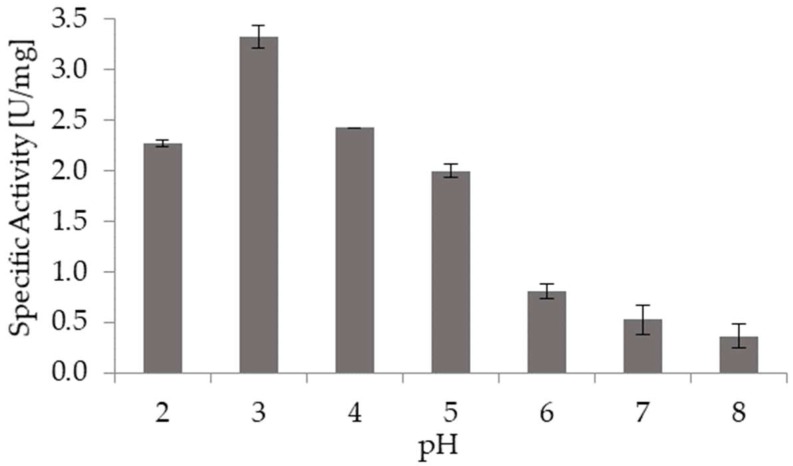
Effect of pH on specific activity of broccoli myrosinase. The bars correspond to the average of three independent experiments and the sticks indicate the standard deviation.

Figure 3 shows the effect of pH on the specific activity of broccoli myrosinase. Myrosinase activity was higher at acidic pH, with the maximum activity reached at pH 3.0. It is remarkable that at pH 2.0 broccoli myrosinase keeps high activity, since this is the stomach pH. Besides, at pH 6.0, which is the condition in small intestine, myrosinase is also active. Thus, if GSL reaches small intestine after the intake of broccoli-derived food, sulforaphane and other isothiocyanates would be the main products that come from the hydrolysis mediated by myrosinase.

### 2.2. Molecular Docking of Broccoli Myrosinase with Substrates and Potential Inhibitors

The molecular docking simulations were carried out at pH 3.0, based on the previous results. The ligands considered in this study correspond to small molecules reported as thioglucosidase inhibitors, and were chosen based on the literature. Table 1 shows the glide scores and docking scores obtained for the 40 myrosinase-ligand complexes. According to Schrödinger program, the docking score (dimensionless) corresponds to the glide score (kcal/mol) modified by the inclusion of Epik state penalties due to protonation (https://www.schrodinger.com/kb/348). To assess the docking of protonated ligands, the docking score should be used. Thus, in this work, docking score was used to compare the stability of the simulated complexes. The average docking score obtained for the potential inhibitors was −5.276, while the docking scores obtained for the substrates sinigrin and glucoraphanin were −5.508 and −6.649, respectively. Then, the myrosinase-glucoraphanin complex is more stable than the myrosinase-sinigrin complex. Among the 40 inhibitors studied, 22 of them had a docking score higher than the average (−5.276). In turn, 17 inhibitors presented a docking score higher than that obtained for sinigrin. However, only arbutin and amygdalin formed a more stable myrosinase complex in comparison with glucoraphanin. The docking scores obtained for amygdalin and arbutin complexes were −6.918 and −7.474, respectively. These values suggest that these compounds would compete with the substrates for the active site of broccoli myrosinase at acidic pH, resulting in more stable complexes and thus preventing the hydrolysis of GSL at that pH. The previous results were confirmed through molecular docking simulations performed in the program Autodock Vina (Table 1). The energy values given by this program agree with the values obtained from Schrödinger simulations, showing the same tendency.

### 2.3. Molecular Interactions

**Figure 4:**
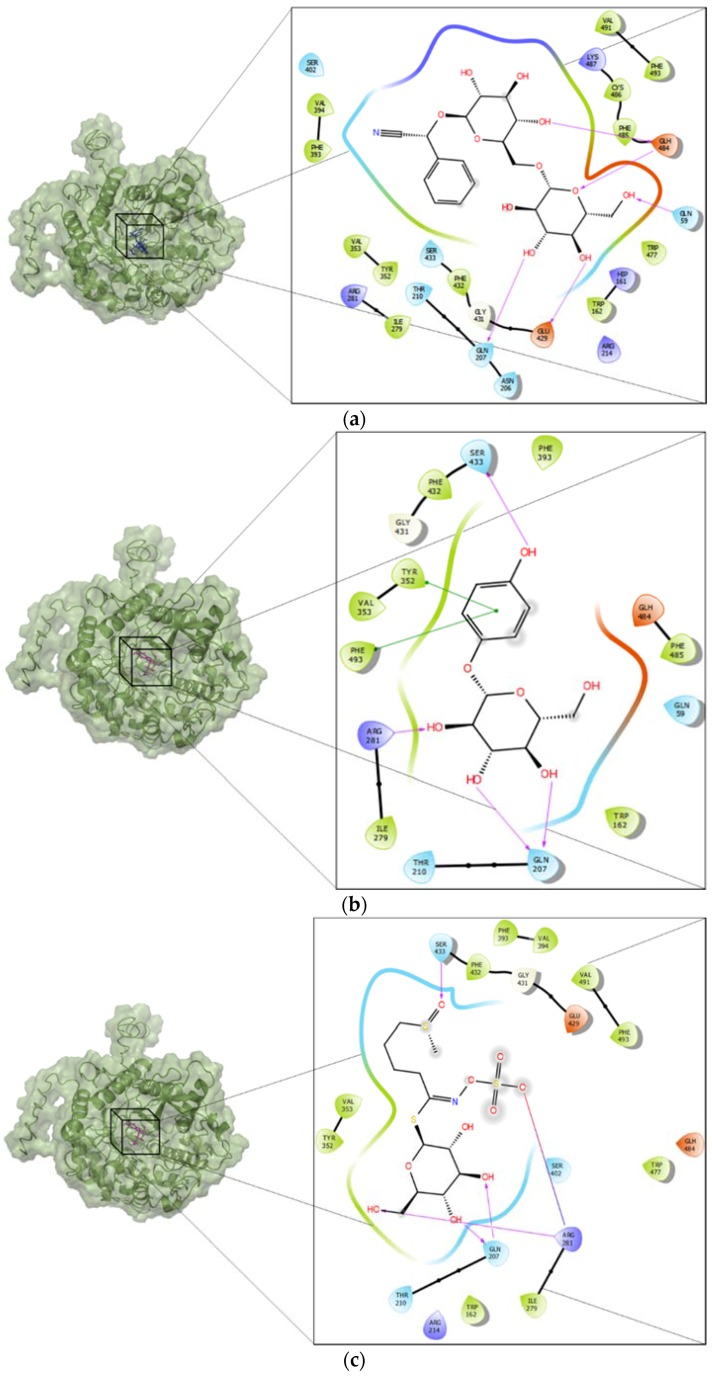

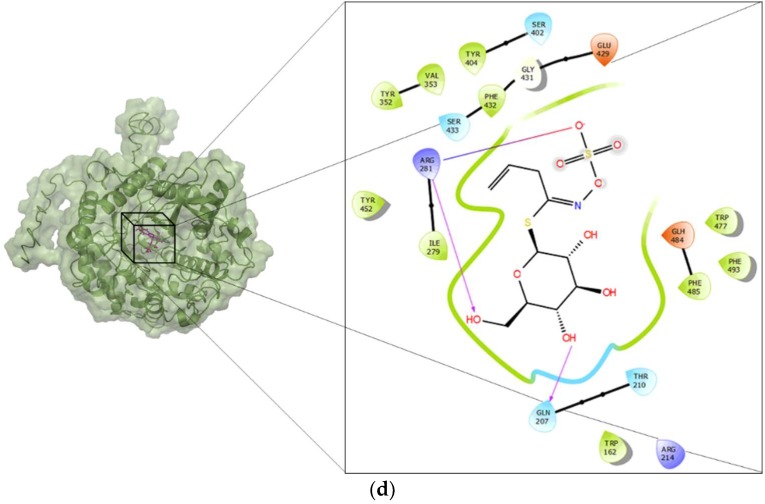
Three-dimensional structure of the complexes between broccoli myrosinase and: (**a**) amygdaline; (**b**) arbutin; (**c**) glucoraphanin; and (**d**) sinigrin.

Docking was performed in a grid centered at the active site of broccoli myrosinase, as suggested in the literature [19]. Figure 4 shows the residues in the active site that interact with arbutin (Figure 4a), amygdalin (Figure 4b), sinigrin (Figure 4c) and glucoraphanin (Figure 4d). The amino acid residues that stabilize amygdalin were Gln207, Glu429, Gln59, and Glh484. Glh484 may act as acid or base. There were hydrophobic zones in the neighborhood of the active site, composed of residues: Val491, Phe493, Phe485, Val394, Phe393, Val353, Phe432, Trp477, Ile279, and Trp162. Additionally, there were positively charged residues, namely Arg281, Arg214, Lys487 and Hip161, the latter being a histidine with different protonation states; and uncharged residues, namely Ser402, Ser433, Thr210 and Asn206. The amino acid residues that stabilize arbutin were Ser433, Tyr352, Phe493, Arg281 and Gln207. The hydrophobic zones were formed by Phe393, Phe432, Val353, Phe485, Trp162, and Ile279. The positively charged residue was Arg281, and uncharged residues were Gln59 and Thr210; and, finally, a glutamic acid, Glh484, with different protonation states. The residues that interact directly with glucoraphanin (Figure 4c) were Ser433, Arg281 and Gln207. There were hydrophobic zones, composed of residues: Phe393, Val394, Phe432, Val491, Phe493, Val353, Tyr352, Trp477, Trp162, and Ile279. Additionally, there were positively charged residues, namely Arg281 and Arg214, and uncharged residues, namely Thr210 and Ser402. Finally, there were Glu429 and Glh484. The latter is a glutamic acid with different protonation states. The residues that interact with sinigrin (Figure 4d) were Arg281 and Gln207. The hydrophobic zones were formed by Tyr404, Val353, Tyr352, Phe432, Tyr452, Ile279, Trp477, Phe493, Phe485, and Trp162. The positively charged residue was Arg214, and uncharged residues were Ser402, Ser433, Gln207 and Thr210. Finally, similar to glucoraphanin, there were Glu429 and Glh484.

## 3. Discussion

The hydrolysis of glucosinolates by myrosinase to form isothiocyanates such as sulforaphane, depends on the chemical conditions. The effect of pH on myrosinase activity is critical, because high myrosinase activity does not imply a high yield of the product of interest due to the formation of undesirable side products. Myrosinase activity strongly depends on pH, whereas the pH that maximizes activity varies between different species, ranging from pH 3 to 8 [20,21].

In the pH stability study, we used sinigrin as substrate because it is one of the most abundant glucosinolates in *Brassica oleracea* species, such as white cabbage, savoy cabbage, red cabbage, kale, Brussels sprouts, cauliflower and kohlrabi, and it is also present in broccoli [22]. Besides, since most authors use sinigrin in myrosinase studies, its use facilitates comparison with literature data. The highest specific activity of broccoli myrosinase and sinigrin was attained at pH 3.0, agreeing with Mahn et al. [18], who reported that myrosinase activity was higher at acidic pH using sinigrin as substrate. Nevertheless, other authors reported that pH below 5 produced a structural destabilization of mustard seeds myrosinase. This would not be the case for broccoli myrosinase. Wasabi myrosinase showed the maximum activity at pH 6.5–7 [23,24].

The results showed that the interaction of myrosinase with amygdaline or arbutin yields complexes of higher stability than those obtained with substrates. For both ligands, the amino acid residues that stabilize the interaction at the active site of myrosinase were Gln207, Glu429, Gln59, Glh484, Ser433, Ser402, and Trp477 for amygdaline and Ser433, Tyr352, Phe493, Arg281 and Gln207 for arbutin. These residues agree with those identified in previous studies about the interaction of myrosinase and glucoraphanin, where molecular docking simulations showed that the residues at the active site responsible for substrate stabilization by hydrogen bonding were Gln207, Ser433, Ser402 and Trp477 [19]. These results suggest that both amygdaline and arbutin interact with broccoli myrosinase at the substrate binding site, behaving as possible competitive inhibitors.

In addition to the aforementioned interactions, in the neighborhood of the active site of broccoli myrosinase, several amino acid residues form a hydrophobic pocket, some of which are highly conserved (Val353, Phe393, Ile279 and Tyr352) and are present in the same position of the polypeptide chain of *Brassica juncea* myrosinase [25]. In this context, it is remarkable that, in addition to interactions by hydrogen bonds and other non-covalent interactions between the ligands and residues of the active site of the enzyme, the hydrophobic interactions between some amino acid residues of the active site with apolar portions of the ligands, can have a very important role in the binding and stabilization of the ligand. Thus, Val394, Phe393, Val353 and Tyr352 are the main residues that interact with the mandelonitrile moiety corresponding to the apolar portion of the amygdalin. In fact, very important evidence supporting this assumption was obtained by Gomez et al. [26], who demonstrated that the enzyme trehalase is strongly inhibited by amygdalin (glucose β-1,6-glucose β-mandelonitrile), but that the gentiobiose (glucose β-1,6-glucose) that lacks the mandelonitrile moiety is not a competitive inhibitor of said enzyme. In the case of arbutin, the main hydrophobic interactions between this ligand and the enzyme occur between residues Tyr352, Val353 and Phe493 with the phenyl moiety of arbutin. For glucoraphanin and sinigrin, the residues involved in these types of interactions are Val353, Tyr352 and Phe432, Tyr452 with the methylsulfinylbutyl and allyl portions of both substrates, respectively. Because both ligands, amygdalin and arbutin, are molecules of higher hydrophobicity than glucoraphanin and sinigrin, it is possible that such differences contribute in part to their greater affinity with the active site of the enzyme, and thus may behave as competitive inhibitors of broccoli myrosinase.

Docking simulations at pH 7 were conducted to elucidate if the selected inhibitors dissociate at that pH, thus enabling myrosinase activity (Table 1). Both programs, Schrödinger and Autodock Vina, gave higher energy values for the myrosinase–inhibitor complexes at pH 7, in comparison with those obtained at pH 3. This suggests that at pH 7 the myrosinase–inhibitor complexes are more unstable and therefore the inhibitor could probably dissociate from the enzyme.

Amygdaline and arbutin are naturally occurring chemical compounds. Amygdalin was initially isolated from bitter almonds (*Prunus dulcis*) in the 1830s, and Laetrile, a semi-synthetic-injectable form of amygdalin, became one of the most popular, non-conventional, anti-cancer treatments in the 1970s [27]. In 1982, a clinical trial, sponsored by the NCI with approval of the US Food and Drug Administration (FDA), failed to demonstrate anticancer activity. Since then, amygdalin has been banned by the FDA and it is not authorized for sale as a drug in the USA or Europe, with some exceptions. Nevertheless, FDA Information is not available about how many people currently consume amygdalin [27].

On the other hand, arbutin is a glycoside extracted from the bearberry plant, which inhibits tyrosinase and thus prevents the formation of melanin. Arbutin has been used in phytotherapy for centuries. Nowadays, arbutin is recognized as a medicinal compound [28]. Accordingly, this compound could be delivered as a nutritional supplement together with myrosinase and glucosinolates, or ingested together with Brassicaceae vegetables, to prevent formation of nitriles and favor sulforaphane bioavailability.

## 4. Materials and Methods

### 4.1. Plant Material

Broccoli (*Brassica oleracea* var. italica) heads were purchased at the local market (Santiago, Chile) from a single supplier. Broccoli florets were immediately processed for protein extraction. Myrosinase was obtained directly from broccoli protein extract, using the protocol described in literature [18].

### 4.2. Myrosinase Activity

Myrosinase activity was assessed using UV-Visible spectrophotometer (Rayleigh UV 1800, Analytical Instrument Co. LTD, Beijing, China) by the method described in literature [19]. Eight hundred microliters of 33-mM sodium phosphate buffer (pH 7.0) and 100 μL of protein extract were preincubated for 3 min at 37 °C, then 100-μL of 100 mM sinigrin was added. Decline in absorbance at 227 nm as a result of sinigrin breakdown was plotted against time, and enzyme activity was calculated from the slope within the linear phase of the graph. One unit of enzyme activity was defined as the amount of myrosinase that catalyzes the hydrolysis of 1 µmol of sinigrin per minute, under the conditions described above. Measurements were made in triplicate and specific activity was expressed as units per milligram of protein.

### 4.3. Effect of pH on Myrosinase Activity

The effect of pH on myrosinase activity was determined in the pH range of 2.0–8.0 using the following buffers: HCl-KCl (pH 2.0), glycine-HCl (pH 3.0), acetate (pH 4.0 and 5.0), sodium phosphate (pH 6.0 and 7.0) and Tris-HCl (pH 8.0), at 37 °C, using a thermostatic bath (Stuart, Staffordshire, UK). Assays were made in triplicate. All reagents were purchased from Sigma-Aldrich, St. Louis, MO, USA.

### 4.4. Molecular Docking

**Figure 5:**
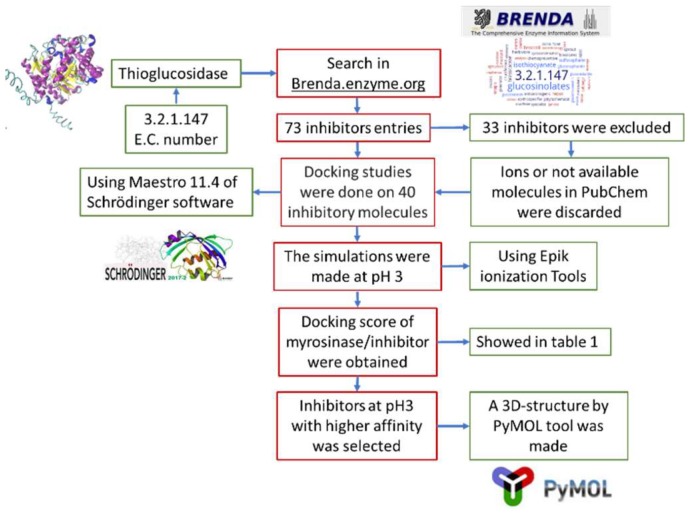
Flowsheet representing the methodology used in the docking simulations.

Figure 5 shows the pipeline used in this work. First, a search in Brenda enzyme database (https://www.brenda-enzymes.org/) was performed, using E.C. number 3.2.1.147, that corresponds to thioglucosidase. This database was chosen because it stores enzymes information obtained from in vitro assays and the data is supported by research papers. Seventy-three entries of inhibitors were obtained, of which 33 were discarded for being ions or because their structure was not available at PubChem (https://pubchem.ncbi.nlm.nih.gov/). Toxic ligands were not discarded because this is a preliminary study that aims to analyze the types of molecules that could be used as myrosinase inhibitors. From that information it would be possible to design a specific inhibitor in the future. The 40 selected ligands were docked on myrosinase (PMDB ID: PM00811093) using Free-Maestro 11.4, of Schrödinger suite [29]. As reference substrates, we used glucoraphanin, which is the most abundant GSL in broccoli, and sinigrin, because it is present in almost all Brassicaceae members and also most studies on myrosinases use sinigrin as substrate. The simulations were conducted at pH 3, using Epik ionization tools [30,31]. The ligands that were proposed as possible myrosinase inhibitors were selected considering that the binding energies of the ligand to broccoli myrosinase were lower than the binding energy of the substrates sinigrin and glucoraphanin. In addition, molecular docking simulations were conducted using AutoDock Vina [32]. Ligands structures were prepared for docking by adding the polar hydrogens and partial charges and defining the rotatable bonds. Myrosinase was also prepared by adding polar hydrogens and merging non-polar hydrogens. Grid map dimensions were assigned to active site residues and to the surrounding surface, according to Roman et al. [19]. Finally, the three-dimensional structure of the chosen complexes was represented by PyMOL tool [33].

#### 4.4.1. Preparation of Broccoli Myrosinase Structure

The three-dimensional structure of broccoli myrosinase was retrieved from Protein Model Data Base (https://bioinformatics.cineca.it/PMDB/) (PMDB ID: PM00811093) [19]. The PDB file of broccoli myrosinase structure was processed with the Protein Preparation Wizard in the Schrödinger suite [34]. The protein structure integrity was adjusted, and the missing side-chain atoms within the protein residues were predicted by Prime. Hydrogen atoms were added after deleting ions, cofactors and water molecules. The protonation and tautomeric states were adjusted to pH 3.0 [35].

#### 4.4.2. Ligand Preparation

**Figure 6:**
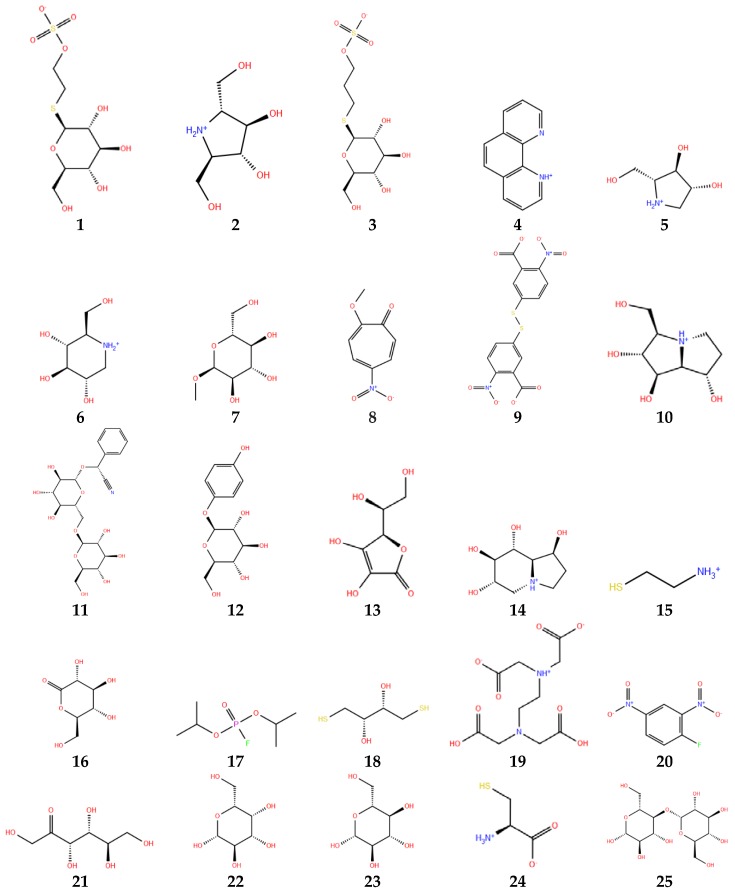

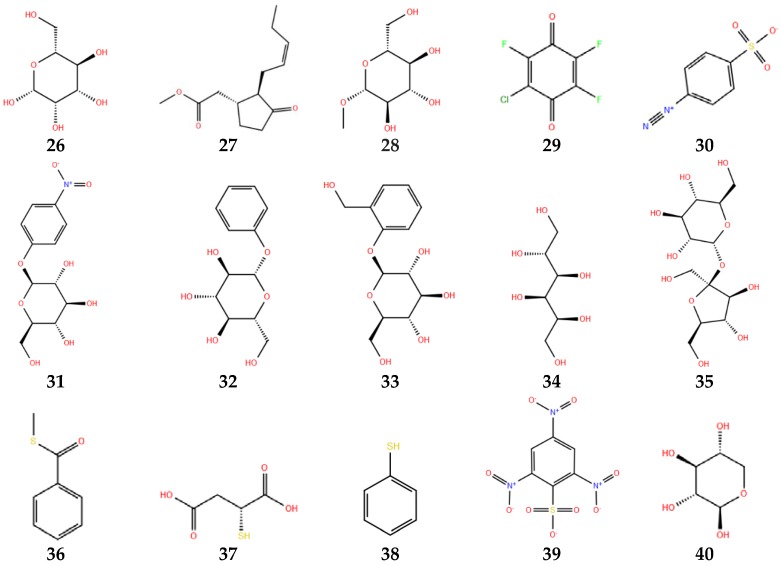
Thioglucosidase inhibitors in protonated state at pH 3. Numbers below each molecule are according to Table 1.

Figure 6 shows the structures of the selected ligands in protonated state. The preparation of the ligands was carried out by means of LigPrep module of Schrodinger Suite [36]. OPLS3 force field was selected for energy minimization. LigPrep converts 2D structures to 3D structures, by adding hydrogens, considering bond lengths and angles, and selecting the conformers structure that shows the lowest conformational energy, which in turn depends on correct chiralities, tautomers, stereochemistries and ring conformations. The ionization state was set at pH range of 3.0 ± 1.0, using EPIK 2.1 ionization tool [35]. All possible protonation states and ionization states, tautomers, stereochemistry, and ring conformations were generated. Stereoisomers were generated with unassigned stereogenic centers, considering a maximum of 32 stereoisomers per ligand. Only the lowest energy conformation was kept for each ligand.

#### 4.4.3. Receptor Grid Generation

Receptor grid was located at the active site of broccoli myrosinase. The grid was a cubic box, centered at the centroid of the active site residues. In glide, the grid was generated keeping the default parameters of van der Waals forces scaling factor 0.08 and charge cut-off 0.15, subjected to OPLS3 force field [37].

#### 4.4.4. Glide Standard Precision (SP) Ligand Docking

Standard precision flexible ligand docking was carried out using glide of Schrödinger [37]. Penalties were applied to non-*cis*/*trans* amide bonds. Van der Waals scaling factor and partial charge cut-off were set at 0.80 and 0.15, respectively, for ligand atoms. Epik state penalties were added to docking score. Final scoring was performed on energy-minimized conformations and displayed as glide score and docking score. The docked conformation with the lowest docking score was recorded for each ligand (Table 1).

#### 4.4.5. Residues that Stabilize Myrosinase-Ligand Complexes

The identification of myrosinase residues that interact with the different ligands in the most stable complex conformations was performed using 2D ligand–protein interaction of Schrödinger suite. In addition, three-dimensional structure of broccoli myrosinase and residue–ligand interactions were visualized with PyMOL tool [33].

## 5. Conclusions

The highest specific activity of broccoli myrosinase was obtained at pH 3.0. Molecular docking analysis suggested that amygdalin and arbutin probably act as broccoli myrosinase inhibitors at acidic pH, since these ligands showed the highest affinity to myrosinase, in comparison with sinigrin and glucoraphanin. Besides, the residues that stabilize the amygdalin and arbutin complexes agree with those that stabilize the substrate (Gln207, Glu429, Tyr352, and Ser433). Accordingly, amygdaline and arbutin would perform as competitive inhibitors of myrosinase at pH 3.0, of which arbutin offers the highest application potential since there is no evidence against its use. At pH 7, the stability of the myrosinase–arbutin complex was lower than at pH 3, suggesting that the interaction is pH-dependent, and that arbutin would probably dissociate at neutral pH. These findings should be verified experimentally in the future.

## Figures and Tables

**Table 1:** Docking scores and glide scores obtained for 40 thioglucosidase inhibitors and two substrates. In parentheses appear the values given by Autodock Vina. The energy values obtained at pH 7 are given in italics.

N°	Inhibitor	PubChem CID *	Glide Score (kcal/mol)	Docking Score
**1**	(2-sulfate) ethyl 1-thio-beta-d-glucopyranoside	23666133	−6.152	−6.152
**2**	(2*R*,5*R*)-dihydroxymethyl-(3*R*,4*R*)-dihydroxypyrrolidine	124702	−5.167	−5.167
**3**	(3-sulfonate) propyl 1-thio-beta-d-glucopyranoside	23674091	−6.392	−6.392
**4**	1,10-phenanthroline	1318	−5.788	−5.782
**5**	1,4-dideoxy-1,4-imino-d-arabinitol	451991	−5.508	−5.508
**6**	1-deoxynojirimycin	29435	−4.671	−4.671
**7**	1-*O*-methyl-alpha-d-glucopyranose	64947	−5.372	−5.372
**8**	2-methoxy-5-nitrotropone	84563	−5.774	−5.148
**9**	5,5′-dithiobis (2-nitrobenzoic acid)	6254	−6.076	−5.984
**10**	Alexine	189,377	−4.778	−4.778
**11**	Amygdalin	656,516	−6.964 (−6.900) *−6.918 (−6.600)*	−6.918
**12**	Arbutin	440,936	−7.842 (−6.900) *−7.474 (−6.500)*	−7.474
**13**	l-ascorbic acid	54,670,067	−5.626	−4.682
**14**	Castanospermine	54,445	−6.426	−6.426
**15**	Cysteamine	6058	−2.981	−2.981
**16**	Delta-gluconolactone	7027	−5.566	−5.566
**17**	Diisopropyl fluorophosphate	5936	−4.601	−4.601
**18**	Dithiothreitol	446,094	−3.613	−3.613
**19**	Ethylenediaminetetraacetic acid	6049	−2.266	−2.170
**20**	Fluorodinitrobenzene	6264	−5.331	−5.331
**21**	Fructose	5984	−4.356	−4.356
**22**	Galactose	6036	−5.671	−5.671
**23**	Glucose	64,689	−6.156	−6.156
**24**	l-Cysteine	5862	−4.176	−4.127
**25**	Maltose	6255	−5.396	−5.396
**26**	Mannose	18,950	−6.239	−6.239
**27**	Methyl jasmonate	5,281,929	−4.966	−4.966
**28**	Methyl-beta-d-glucopyranoside	445,238	−5.787	−5.787
**29**	Monochlorotrifluoro-*p*-benzoquinone	53,662,935	−5.092	−5.092
**30**	*p*-diazabenzenesulfonic acid	67,540	−4.707	−4.594
**31**	*p*-nitrophenyl-beta-d-glucopyranoside	92,930	−5.842	−5.842
**32**	Phenyl-beta-d-glucopyranoside	65,080	−6.524	−6.524
**33**	Salicin	439,503	−5.950	−5.950
**34**	Sorbitol	5780	−4.013	−4.013
**35**	Sucrose	5988	−5.305	−5.305
**36**	Thiobenzoate	80,024	−5.088	−5.088
**37**	Thiomalate	5,352,130	−5.475	−4.782
**38**	Thiophenol	7969	−4.786	−4.786
**39**	Trinitrobenzenesulfonic acid	11,045	−5.470	−5.470
**40**	Xylose	135,191	−6.164	−6.164
**Mean glide and docking score**		**---**	**−5.341**	**−5.276**
**Sinigrin**		23,682,211	−5.508	−5.508
**Glucoraphanin**		9,548,634	−6.649	−6.649

* Compound identifier.
